# A New Mode of Arresting Nasal and Uterine Hæmorrhage

**Published:** 1878-08

**Authors:** Edward C. Huse

**Affiliations:** Rockford, Ills.


### Clinical Reports.

### NOTES FROM PRIVATE PRACTICE.

### *A New Mode of Arresting Nasal and Uterine Hemorrhage.*

It is a wise maxim that the simplest things are best.

An ordinary rubber condom is passed into the anterior nares, by means of a flexible catheter, and carried back, along the floor of the palate, until visible behind the velum pendulum palati. A few pieces of pounded ice are then introduced into it and pushed back also. Thus the temperature is lowered, locally The elastic rubber bag is then inflated with air, by gathering its neck tightly around the nozzle of a bulbous syringe, till the nasal cavity is filled, the elasticity of the bag adapting its shape to the turbinated bones and septum nasi, and the hæmorrhage, under the combined influence of cold and pressure, of course ceases. Bellocq's sound for tamponing is not always accessible, and even if it were, this plan is better. It possesses another advantage over the sponge, which is used with Bellocq's sound, and that is it never can irritate, is more cleanly, and if renewed bleeding occur, on allowing the air or water to escape, one need only refill it with ice or water, leaving it still *in situ*, till the hæmorrhage is controlled by constitutional measures.

It is almost needless to say that the promptest and most potent of these is a hypodermic exhibition of one gram of the extract of ergot, which is prepared in a soluble form by Squibb.

In uterine hemorrhage, or rectal, I have not yet used this tampon, but its entire adaptation in these cases is obvious. The principle involved is somewhat similar to the uterine dilators of Barnes, though not identical with it, for in those the object is to overcome muscular contractility; in this, to cause the coagulum or stasis which *cold* combined with elastic and equable pressure produce.

EDWARD C. HUSE, M. D.

ROCKFORD, ILLS., June 24th, 1878.

(We are pleased to publish the practical communication of our correspondent, but must advise him that his method of procedure has been given to the public by an earlier writer.—ED.)

